# The Evaluation of Antioxidant and Anti-Inflammatory Effects of *Eucommia ulmoides* Flavones Using Diquat-Challenged Piglet Models

**DOI:** 10.1155/2017/8140962

**Published:** 2017-08-15

**Authors:** Daixiu Yuan, Tarique Hussain, Bie Tan, Yanhong Liu, Peng Ji, Yulong Yin

**Affiliations:** ^1^Department of Medicine, Jishou University, Jishou, Hunan 416000, China; ^2^National Engineering Laboratory for Pollution Control and Waste Utilization in Livestock and Poultry Production, Key Laboratory of Agro-Ecological Processes in Subtropical Region, Institute of Subtropical Agriculture, Chinese Academy of Sciences, Changsha, Hunan 410125, China; ^3^University of Chinese Academy of Sciences, Beijing 100008, China; ^4^Department of Animal Science, University of California Davis, Davis, CA 95616, USA

## Abstract

This study was designed to evaluate the antioxidant and anti-inflammatory effects of *Eucommia ulmoides* flavones (EUF) using diquat-challenged piglet models. A total of 96 weaned piglets were randomly allotted to 1 of 3 treatments with 8 replication pens per treatment and 4 piglets per pen. The treatments were basal diet, basal diet + diquat, and 100 mg/kg EUF diet + diquat. On day 7 after the initiation of treatment, the piglets were injected intraperitoneally with diquat at 8 mg/kg BW or the same amount of sterilized saline. The experiment was conducted for 21 days. EUF supplementation improved the growth performance of diquat-treated piglets from day 14 to 21. Diquat also induced oxidative stress and inflammatory responses and then impaired intestinal morphology. But EUF addition alleviated these negative effects induced by diquat that showed decreasing serum concentrations of proinflammatory cytokines but increasing antioxidant indexes and anti-inflammatory cytokines on day 14. Supplementation of EUF also increased villi height and villous height, crypt depth, but decreased the histopathological score and MPO activity compared with those of diquat-challenged pigs fed with the basal diet on day 14. Results indicated that EUF attenuated the inflammation and oxidative stress of piglets caused by diquat injection.

## 1. Introduction

Oxidative stress is a common phenomenon in humans and animals that resulted from a large number of biological and environmental factors and stressors [1]. Under normal physiological conditions, there is a balance between the production of oxidants and antioxidants in the biological system [2]. The overwhelming of free radicals could impair this redox balance and thereby results in oxidative stress, including the oxidation of proteins, lipids, and nucleic acids [3, 4]. Many chronic diseases have been reported to be linked with the excessive production of reactive oxygen species (ROS) [5, 6].

Natural compounds present in plants have been reported to exhibit antioxidant activities that interact with ROS/reactive nitrogen species (RNS) and thus terminate chain reaction [7–9]. *Eucommia ulmoides* (EU) (also known as “Du Zhong” in Chinese) contains enriched chemical components such as lignins, iridoids, phenolics, steroids, and flavonoids and, therefore, presents various medicinal properties as a Chinese traditional medicine [8, 10]. The leaf of EU also contains abundant secondary metabolites, such as flavonoids [8, 11, 12]. It has been reported that flavonoids have strong antioxidant activities; it shows direct scavenging free radicals, suppression of proinflammatory cytokines through inhibition of reactive oxygen species and nitric oxide, decreasing inflammatory genes including cyclooxygenases (COXs) and inducible nitric oxide synthase (iNOS), upregulating antioxidant enzymes, modulating transcription factors such as NF-*κ*B and AP-1, and enhancing the Nrf2 signaling pathway [13–16].

Therefore, the objectives of this experiment were to investigate the antioxidant activities and anti-inflammatory effects of the flavones extracted from the leaves of EU using an oxidative stress piglet model induced by diquat that is a bipyridyl herbicide exerts ability to produce free radicals by redox-cycling metabolism and widely accepted in vivo model of oxidative stress [17–19].

## 2. Materials and Methods

### 2.1. EUF Extract

EUF extraction and total flavone content determination were conducted according to the methods of Li et al. [20, 21] at the Department of Medicine, Jishou University (Jishou, Hunan, China). The leaves of EU were shade dried and finely powdered. The extraction was performed using 65% ethanol in Erlenmeyer flasks for 30 min using an ultrasonic cleaner at 50°C, followed by filtration. This extraction process was repeated twice. The filtrate was then concentrated using a rotary vacuum evaporator at 70°C. The concentrated extract solution was dipping degreased using petroleum ether and then purified using a macroporous resin by rinsing the column with distilled water and static absorption for 2 h. The fraction was eluted with the 90% ethanol for 2 h and then sequentially concentrated, washed twice with isopropanol, filtrated, concentrated, and lyophilized. The content of the total flavones in EUF powder was 83.61% analyzed by ultraviolet spectrophotometer methods using rutin as calibration standard [22].

### 2.2. Animals and Experimental Design

The animal experiments were approved by the Institutional Animal Care and Use Committee of the Institute of Subtropical Agriculture, Chinese Academy of Sciences (2013020).

A total of 96 three-breed crossbred (Duroc × Landrace × Large Yorkshire) piglets weaned at 21 days were randomly assigned to receive 1 of 3 treatments with 8 replicate pens/treatment and 4 piglets/pen. The 3 treatments include basal diet, basal diet + diquat, and 100 mg/kg EUF diet + diquat. The diets were formulated to meet the nutrient requirements for weanling piglets (Table 1). Diquat was purchased from Sigma-Aldrich (St. Louis, MO, USA) and a dose of 8 mg/kg BW was used according to the results reported by Yin et al. [18]. The piglets were housed individually in an environmentally controlled nursery room with hard plastic-slatted flooring. All animals had free access to water. After a 7-day adaptation period, piglets were fed with their respective diets 3 times per day at 8:00, 13:00, and 18:00 for a 21-day period. All piglets were weighed weekly, their average daily gain, daily feed intake, and gain : feed ratio were calculated throughout the entire experiment.

On day 7, the piglets in basal diet + diquat and EUF diet + diquat treatments received an intraperitoneal injection of diquat at 8 mg/kg BW while the piglets on basal diet received the same volume of sterilized saline. On day 14 and 21, 8 piglets (1 pig/pen) were randomly selected and blood samples were collected aseptically from the jugular vein at 2 h after a.m. feeding. Serum samples were obtained by centrifuging blood samples at 2000 ×g for 10 min at 4°C and then immediately stored at −80°C for further analysis. Piglets were anesthetized with sodium pentobarbital and euthanized by jugular puncture. The intestinal samples were collected from the jejunum, ileum, anterior colon, and posterior colon and fixed in 4% formaldehyde for morphology analysis and histopathological grading. Anterior and posterior colonic tissues were immediately snap-frozen in liquid N and stored at −80°C for myeloperoxidase (MPO) activity analysis.

### 2.3. Detection of Antioxidative Capacity

Serum concentrations of superoxide dismutase (SOD), glutathione peroxidase (GSH-Px), catalase (CAT), malondialdehyde (MDA), total antioxidant capacity (T-AOC), and GSH were measured using their corresponding assay kits (Nanjing Jiancheng, Nanjing, China) according to manufacturer instructions. In brief, SOD, CAT, and GSH-Px were analyzed by xanthine oxidase-xanthine reaction method, CAT-H_2_O_2_ reaction method, and reduced glutathione method, respectively. MDA capacity was assayed by 2-thiobarbituric acid method and T-AOC was detected by ferric-reducing/antioxidant power reaction method. All samples were measured by UV/visible spectrophotometer (UV-2450, Shimadzu, Kyoto, Japan).

### 2.4. Analysis of Serum Concentrations of Cytokines

Serum concentrations of interleukin (IL)-1*β*, IL-4, IL-6, IL-8, IL-12, granulocyte macrophage colony-stimulating factor (GM-CSF), transforming growth factor-beta 1 (TGF-*β*1), tumor necrosis factor- (TNF-) *α*, IL-10, and interferon-gamma (IFN-*γ*) were determined by using Porcine Cytokine Array QAP-CYT-1 (RayBiotech Inc., Guangzhou, China). An array-based multiplex ELISA system was used for quantitative measurement of multiple cytokines according to the manufacturer's protocol. Briefly, 100 *μ*L of sample diluent was added to each well for 30 min to block slides and then decanted. 100 *μ*L of the sample or cytokine standard was added to the plate and incubated overnight at 4°C. The samples were decanted and washed 5 and 2 times with Wash Buffers I and II, respectively. The plate was incubated in 80 *μ*L of the detection antibody cocktail for 2 h and then washed as before. 80 *μ*L Cy3 dye equivalent dye-conjugated streptavidin was added to each well and the plate was incubated in a dark room for 1 h. After being washed 5 times, the slides were placed in the slide washer/dryer and gently washed with Wash Buffers I and II for 15 and 5 min, respectively. The signals were visualized using InnoScan 300 Microarray Scanner (Innopsys, Parc d'Activités Activestre, Carbonne, France) equipped with a Cy3 wavelength (green channel, at an excitation of 532 nm), and the quantitative data analysis was performed using the Quantibody® Q-Analyzer (QAP-CYT-1, RayBiotech Inc.).

### 2.5. Determination of Serum Diamine Oxidase (DAO) and D-Lactate

The reaction system for the serum concentration of DAO determination included 0.1 mL (4 *μ*g) horseradish peroxidase solution (Sigma-Aldrich, St. Louis, USA), 3 mL PBS (0.2 M, pH 7.2), 0.1 mL O-dianisidine methanol solution (500 *μ*g of O-dianisidine) (Sigma-Aldrich, St. Louis, USA), 0.5 mL sample, and 0.1 mL substrate solution (175 *μ*g of cadaverine dihydrochloride) (Sigma-Aldrich, St. Louis, USA). The processed samples were incubated in an incubator chamber at 37°C for 30 min and measured at 436 nm by UV/visible spectrophotometer-UV-2450 (Shimadzu, Kyoto, Japan) [23]. Serum D-lactate was determined using a D-Lactate Assay Kit (BioVision, Mountain View, San Francisco, USA) in accordance with the manufacturer's instruction [24].

### 2.6. Intestinal Morphology Evaluation and Histopathological Grading

The jejunal and ileal morphologies were analyzed using hematoxylin eosin staining according to Xiao et al. [23]. Villous height and crypt depth were measured with computer-assisted microscopy (Micrometrics TM; Nikon ECLIPSE E200, Tokyo, Japan).

Histopathological grading of the jejunum, ileum, anterior colon, and posterior colon was performed as described previously [25]. Histological scoring was carried out by a veterinary pathologist using the methods of Huang et al. [25] that ranged from 0 (minimal injury) to 15 (maximal injury) corresponding to four grades that included mononuclear or polymorphonuclear cell infiltration, histological injury, and erosion or epithelial hyperplasia.

### 2.7. Analysis of Myeloperoxidase (MPO) Activity in the Colon

Colon samples were homogenized in 10 volumes of ice-cold potassium phosphate buffer (pH 6.0) containing 0.5% hexadecyltrimethylammonium hydroxide through a high-pressure homogenizer at 10,000–15,000 rpm at 4°C. The homogenate was centrifuged at 2500 × *r* at 4°C for 15 min and the supernatant was transferred into PBS (pH 6.0) containing 0.17 mg/mL 3, 3′-dimethoxybenzidine and 0.0005% H_2_O_2_. MPO activity was assessed by measuring the H_2_O_2_-dependent oxidation of 3, 3′-imethoxybenzidine. One unit of enzyme activity is defined as the amount of MPO present that causes a change in absorbance per min at 460 nm and 37°C [26, 27].

### 2.8. Statistical Analysis

The data of growth performance were performed with an analysis of variance (ANOVA) for repeated measures and others were subjected to ANOVA using SPSS 17.0 software (SPSS Inc., Chicago, IL, USA). The differences among treatments were evaluated using Tukey's test. Probability values < 0.05 were taken to indicate statistical significance.

## 3. Results

### 3.1. Growth Performance

The body weight, average daily gain, average daily feed intake, and gain : feed ratio are shown in Table 2. The body weight on days 0, 7, and 14 were similar among the treatments (*p* > 0.05). The body weight of piglets in basal diet + diquat treatment were lighter (*p* < 0.05) than those in the basal diet and EUF diet + diquat treatments on day 21. Compared with the piglets in basal diet treatment, diquat exposure reduced average daily gain and gain : feed ratio from day 7 to 14 and day 14 to 21, as well as average daily feed intake and gain : feed ratio from day 14 to 21 (*p* < 0.05). However, there were no difference in the average daily gain, average daily feed intake, and gain : feed ratio between piglets of basal diet treatments and EUF diet + diquat treatment (*p* > 0.05). In diquat-treated piglets, dietary EUF increased the average daily gain from day 14 to 21, average daily feed intake from day 7 to 14 and day 14 to 21, and gain : feed ratio from day 7 to 14 (*p* < 0.05).

### 3.2. Serum Antioxidant Parameters

On day 14, exposure to diquat decreased (*p* < 0.05) the serum concentrations of SOD, GSH-Px, CAT, T-AOC, and GSH in piglets of basal diet treatments. But the supplementation of EUF increased (*p* < 0.05) serum concentrations of SOD, GSH-Px, CAT, T-AOC, and GSH in piglets in EUF diet + diquat treatment compared with those in pigs in the basal diet + diquat treatment. On day 21, no differences (*p* > 0.05) were observed in the serum concentration of SOD, CAT, T-AOC, and GSH among treatments, with the exception that pigs in EUF treatment had greater (*p* < 0.05) GSH-Px than diquat-challenged pigs fed with the basal diet. There was no difference in the serum concentration of MDA among treatments on days 14 and 21 (*p* > 0.05) (Table 3).

### 3.3. Serum Profiles of Cytokines

Exposure to diquat increased (*p* < 0.05) serum concentrations of IL-1*β*, IL-6, IL-8, IL-12, GM-CSF, TNF-*α*, IL-10, and IFN-*γ* but decreased (*p* < 0.05) TGF-*β*1 content when pigs were fed with the basal diet on day 14. Dietary EUF decreased (*p* < 0.05) the serum concentrations of IL-1*β*, IL-6, IL-8, IL-12, GM-CSF, TNF-*α*, IL-10, and IFN-*γ* but increased (*p* < 0.05) serum IL-4 and TGF-*β*1 compared with diquat-challenged pigs fed with the basal diet on day 14. On day 21, no differences (*p* > 0.05) were observed in serum cytokine concentrations among 3 treatments, except that diquat injection increased (*p* < 0.05) serum TNF-*α* and IL-10 compared with nonchallenged pigs fed with the basal diet. Supplementation of EUF reduced (*p* < 0.05) serum TNF-*α* concentration when pigs were challenged with diquat (Table 4).

### 3.4. Serum Concentrations of D-Lactate and Diamine Oxidase

Diquat exposure increased (*p* < 0.05) serum concentrations of D-lactate and diamine oxidase in pigs fed with the basal diet on day 14; however, this was not the case on day 21. No differences were observed in the serum concentration of D-lactate and diamine oxidase between pigs fed with the basal diet and the EUF diet on day 14 and 21 (*p* > 0.05) (Table 5).

### 3.5. Jejunal and Ileal Morphology

Diquat challenge reduced (*p* < 0.05) jejunal and ileal villi height and jejunal and ileal villous height, crypt depth, but increased (*p* < 0.05) ileal crypt depth on day 14 when pigs were fed the basal diet. Inclusion of EUF increased (*p* < 0.05) jejunal and ileal villi height and villous height, crypt depth, of diquat-challenged pigs compared with those in the basal diet on day 14. No differences were observed in villous height and crypt depth of jejunum and ileum among 3 treatments on day 21 (*p* > 0.05) (Table 6).

### 3.6. Histopathological Grading

Diquat exposure increased (*p* < 0.05) the histopathological grading of the jejunum, ileum, anterior colon, and posterior colon on day 14, but no differences (*p* > 0.05) were observed in histopathological grading on day 21. Compared with that in basal diet + diquat treatment, lower histopathological grading of the jejunum, ileum, anterior colon, and posterior colon of piglets in EUF + diquat treatment on day 14 were observed (*p* < 0.05) (Table 7).

### 3.7. Myeloperoxidase Activity

**Figure 1:**
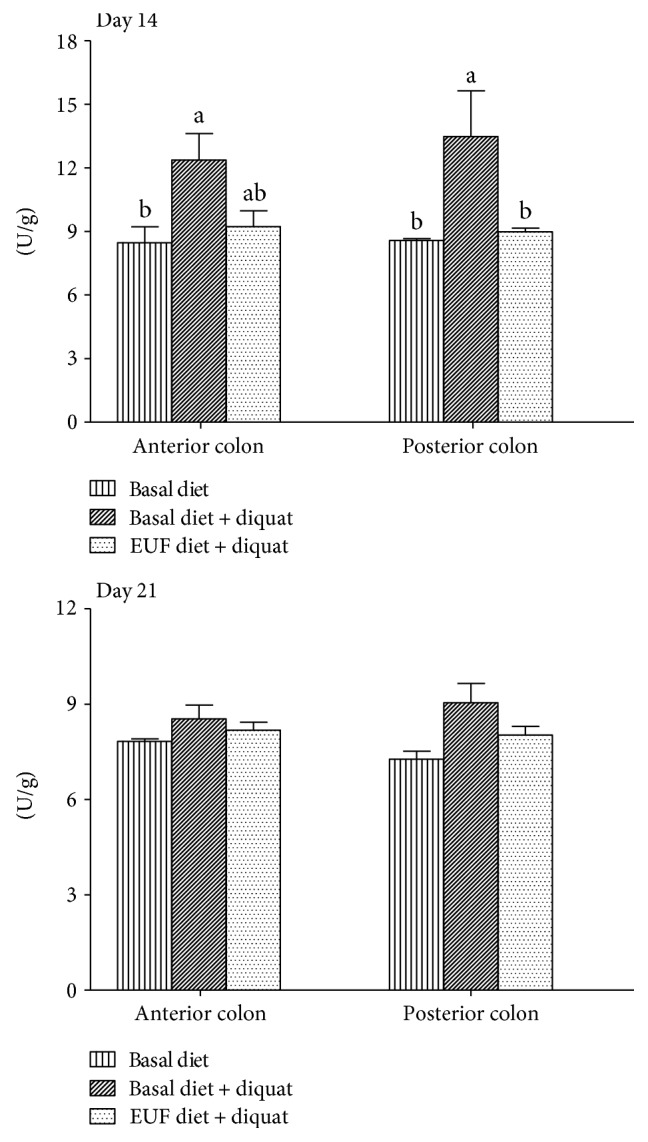
Myeloperoxidase activity in the colon of piglets. Values are the mean ± SEM, *n* = 8 per treatment group. ^a-b^Mean values sharing different superscripts within anterior colon or posterior colon differ (*p* < 0.05).

Diquat challenge increased (*p* < 0.05) the MPO activity in the anterior and posterior colon of piglets on day 14 if they were fed with the basal diet. Dietary EUF supplementation reduced (*p* < 0.05) the MPO activity in the posterior colon compared with diquat-challenged pigs fed with the basal diet. However, no difference was observed in the MPO activity among 3 treatments on day 21 (*p* > 0.05) (Figure 1).

## 4. Discussion

Oxidative stress which resulted in cellular injury and tissue damage has been increasingly recognized as a contributing factor in many chronic diseases such as heart disease, Alzheimer's and Parkinson's diseases, and even cancer [1, 5]. Therefore, inhibition of oxidative stress will be a potential strategy to prevent chronic diseases. There has been considerable interest in the isolation and characterization of antioxidative agents from natural products [6, 8, 9]. The present study is focusing on the antioxidative activity of flavones in the EU leafs that are widely cultivated in China.

In the current experiment, oxidative stress piglet model induced by diquat was used and has been widely used *in vivo* [18, 19]. Diquat has been reported to impair growth performance and nutrient utilization [18, 19]. The reduced gut morphology and growth performance by diquat challenge in the present are in agreement with published report [28]. This is mainly due the disruption in the oxidative balance [18], which is evidenced by the decrease in serum concentrations of SOD, GSH-Px, CAT, T-AOC, and GSH after exposure to diquat injection in piglets of basal diet treatments. In the previous studies, diquat has been demonstrated to increase serum MDA concentration but also to inhibit the activities of SOD and GSH-Px [18, 19]. In addition, the present results of serum cytokine concentrations, intestinal histopathological grading, and MPO activity indicated that diquat evaluates the inflammatory response of weaned pigs, which is consistent with previous research [9, 28, 29].

However, dietary supplementation with EUF showed to alleviate these negative effects induced by diquat. Firstly, EUF significantly elevated the growth performance of weaned piglets and this growth promotion effect of flavonoids also demonstrated in geese, ducks, meat sheep, and fatting pig [30–33]. Therefore, a large scale of performance trial should be conducted to verify the potential of using EUF as a growth promoter for weaned pigs.

The mechanisms of the antioxidant capacity of flavones include suppression of ROS formation by either inhibition of enzymes involved in their production, scavenging of ROS, or upregulation or protection of antioxidant defenses [8]. Diquat could stimulate cellular production of ROS and inhibit the activities of antioxidant enzymes such as SOD and GSH-Px [18, 19, 34]. Antioxidant enzymes including SOD, CAT, and GSH-Px can neutralize toxic oxygen products, thereby normalizing the body homeostasis [2]. SOD converts superoxide radicals into H_2_O_2_, which is converted into water by GSH-Px and CAT [35]. Oxidative stress results from the failure of antioxidant enzymes to eliminate free radicals [36]. The present results indicated that EUF exerted antioxidant effects at the first line of defense by increasing the level of antioxidant enzymes in the blood to neutralize ROS.

The relationship between oxidative stress and inflammation has been documented by previous research [5, 37]. In the present study, dietary supplementation with EUF significantly alleviated the inflammatory response induced by diquat. Excess antioxidant enzymes in the serum and intestine may be associated with the production of proinflammatory and anti-inflammatory cytokines in the respective locations [38]. Evidences have shown that free radicals especially H_2_O_2_ and NO may respond as secondary messengers to stimulate proinflammatory cytokines [39, 40]. Serum cytokine profiles could be a used as markers of inflammation [41, 42]. Proinflammatory cytokines such as IL-1*β*, IL-6, IFN-*γ*, TNF-*α*, and IL-1 are released by macrophages upon activation of the NF-*κ*B pathway, a crucial factor responsible for inducing inflammatory response in pathological conditions [43, 44].

It has demonstrated that flavonoids were not absorbed well and their concentrations could be much higher in the lumen of the gastrointestinal tract than were ever achieved in plasma [45]. Therefore, the digestive tract is the major site of antioxidant defense afforded by flavonoids [45, 46]. The EUF improved the morphological structure and barrier function of the intestine in the present study, which is evidenced by higher villous height and lower concentrations of D-lactate and diamine oxidase. As the markers of intestinal integrity, lower serum diamine oxidase and D-lactate contents released from upper villi of the small intestine showed smaller damage of intestinal integrity [47, 48]. The improvement in intestinal barrier function may reflect the alleviation of inflammatory response [49].

In conclusion, flavones extracted from *Eucommia ulmoides* leaf have shown antioxidative activity and anti-inflammatory effects. The results indicated that dietary supplementation with EUF alleviated the growth performance impairment, oxidative stress, inflammatory response, and intestinal damage induced by diquat in piglets. These findings will be helpful for the development of future antioxidant therapeutics and new anti-inflammatory drugs and the application of EUF in piglets. Further studies will be needed to elucidate the molecular mechanisms of EUF-regulating oxidative stress in inflammatory disease.

## Figures and Tables

**Table 1:** Composition of basal diets (as-fed basis).

Ingredients (%)		Chemical composition	
Corn	59.00	Calculated digestible energy, kcal/kg	3510.53
Soybean meal	9.00	Dry matter	84.6
Extruded soybean	8.00	Crude protein	17.5
Fermented soybean meal	5.00	Calcium	0.54
Fish meal	3.00	Total phosphorus	0.68
Whey powder	8.00	Lysine	1.51
Soybean oil	2.00	Methionine	0.39
Sucrose	2.00	Threonine	0.86
Premix^a^	1.00	Tryptophan	0.20
Calcium citrate	0.60		
Calcium hydrogen phosphate	1.00		
Salt	0.30		
98% lysine	0.64		
Threonine	0.15		
Methionine	0.10		
Compound acidifiers	0.21		

^a^Providing the following amounts of vitamins and minerals per kilogram on an as-fed basis: Zn (ZnO), 50 mg; Cu (CuSO_4_), 20 mg; Mn (MnO), 55 mg; Fe (FeSO_4_), 100 mg; I (KI), 1 mg; Co (CoSO_4_), 2 mg; Se (Na_2_SeO_3_), 0.3 mg; vitamin A, 8255 IU; vitamin D3, 2000 IU; vitamin E, 40 IU; vitamin B1, 2 mg; vitamin B2, 4 mg; pantothenic acid, 15 mg; vitamin B6, 10 mg; vitamin B12, 0.05 mg; nicotinic acid, 30 mg; folic acid, 2 mg; vitamin K3, 1.5 mg; biotin, 0.2 mg; choline chloride, 800 mg; and vitamin C, 100 mg. The premix did not contain additional copper, zinc, antibiotics, or probiotics.

**Table 2:** Growth performance of piglets.

Item	Basal diet	Basal diet + diquat	EUF diet + diquat	*p* value
Body weight (kg)				
Day 0	6.48 ± 0.32	6.50 ± 0.29	6.49 ± 0.32	0.999
Day 7	8.51 ± 0.35	8.53 ± 0.31	8.52 ± 0.36	0.999
Day 14	10.87 ± 0.19	10.12 ± 0.41	10.64 ± 0.28	0.232
Day 21	14.15 ± 0.35^a^	12.51 ± 0.45^b^	13.89 ± 0.34^a^	0.014
Average daily gain (g/d)				
Day 0 to 7	290.51 ± 6.21	290.17 ± 5.11	290.44 ± 10.46	0.999
Day 7 to 14	336.57 ± 17.47^a^	226.95 ± 27.36^b^	302.26 ± 19.46^a, b^	0.006
Day 14 to 21	468.55 ± 21.35^a^	342.14 ± 28.26^b^	464.71 ± 25.42^a^	0.002
Average daily feed intake (g/d)				
Day 0 to 7	293.45 ± 31.21	300.13 ± 16.31	299.56 ± 10.46	0.970
Day 7 to 14	389.01 ± 12.67^a^	308.17 ± 27.78^b^	351.16 ± 19.43^a^	0.041
Day 14 to 21	680.56 ± 27.43^a, b^	599.05 ± 32.03^b^	706.63 ± 25.65^a^	0.037
Gain : feed (g/g)				
Day 0 to 7	0.99 ± 0.01	0.97 ± 0.02	0.97 ± 0.02	0.647
Day 7 to 14	0.87 ± 0.02^a^	0.74 ± 0.05^b^	0.86 ± 0.02^a^	0.020
Day 14 to 21	0.69 ± 0.02^a^	0.57 ± 0.04^b^	0.66 ± 0.02^a, b^	0.018

^a^Values are the mean ± SEM, *n* = 8 per treatment group. ^a-b^Mean values sharing different superscripts within a row differ (*p* < 0.05).

**Table 3:** Serum concentrations of superoxide dismutase, glutathione peroxidase, catalase, malondialdehyde, total antioxidant capacity, and glutathione in piglets.

Item	Basal diet	Basal diet + diquat	EUF diet + diquat	*p* value
Day 14				
Superoxide dismutase, U/mL	104.21 ± 2.12^a^	73.51 ± 6.31^b^	94.87 ± 3.37^a^	<0.001
Glutathione peroxidase, U/mL	359.34 ± 3.12^a^	335.21 ± 4.14^b^	360.85 ± 2.49^a^	<0.001
Catalase, U/mL	7.02 ± 0.21^a^	5.73 ± 0.38^b^	6.97 ± 0.38^a^	0.019
Malondialdehyde, nmol/mL	4.59 ± 0.24	5.76 ± 0.78	4.48 ± 0.36	0.175
Total antioxidant capacity, U/mL	1.25 ± 0.04^a^	0.54 ± 0.01^b^	1.14 ± 0.10^a^	<0.001
Glutathione, mg/L	3.24 ± 0.10^a^	2.43 ± 0.09^b^	3.01 ± 0.15^a^	<0.001
Day 21				
Superoxide dismutase, U/mL	92.18 ± 3.24	82.15 ± 2.16	89.47 ± 4.31	0.116
Glutathione-peroxidase, U/mL	324.25 ± 4.46^a, b^	314.49 ± 5.68^b^	334.15 ± 4.41^a^	0.033
Catalase, U/mL	7.96 ± 0.57	6.97 ± 0.49	7.25 ± 0.68	0.480
Malondialdehyde, nmol/mL	5.42 ± 0.18	5.67 ± 0.49	5.47 ± 0.47	0.900
Total antioxidant capacity, U/mL	1.14 ± 0.08	0.98 ± 0.09	1.21 ± 0.11	0.232
Glutathione, mg/L	3.18 ± 0.15	2.87 ± 0.21	3.14 ± 0.34	0.633

Values are the mean ± SEM, *n* = 8 per treatment group. ^a-b^Mean values sharing different superscripts within a row differ (*p* < 0.05).

**Table 4:** Serum profiles of cytokines in piglets.

Item	Basal diet	Basal diet + diquat	EUF diet + diquat	*p* value
Day 14 (pg/ml)				
IL-1*β*	287.23 ± 8.41^c^	847.24 ± 31.47^a^	387.47 ± 15.46^b^	<0.001
IL-4	294.53 ± 20.14^b^	286.41 ± 19.54^b^	564.56 ± 35.64^a^	<0.001
IL-6	21.25 ± 1.34^c^	257.24 ± 13.24^a^	54.36 ± 8.68^b^	<0.001
IL-8	105.45 ± 12.54^b^	975.64 ± 60.89^a^	243.57 ± 30.42^b^	<0.001
IL-12	146.41 ± 9.57^b^	345.48 ± 35.54^a^	208.65 ± 24.58^b^	<0.001
GM-CSF	124.17 ± 10.58^b^	240.58 ± 27.58^a^	138.42 ± 16.65^b^	<0.001
TGF-*β*1	954.24 ± 23.42^a^	514.35 ± 42.52^b^	895.44 ± 28.56^a^	<0.001
TNF-*α*	1.56 ± 0.21^b^	124.25 ± 23.56^a^	34.45 ± 6.54^b^	<0.001
IL-10	0.45 ± 0.01^b^	1.24 ± 0.25^a^	0.68 ± 0.08^b^	0.004
IFN-*γ*	0.06 ± 0.01^b^	0.58 ± 0.11^a^	0.21 ± 0.05^b^	<0.001
Day 21 (pg/ml)				
IL-1*β*	221.56 ± 11.25	356.45 ± 32.54	215.46 ± 20.56	0.417
IL-4	256.25 ± 25.54	248.36 ± 20.89	324.12 ± 40.56	0.174
IL-6	53.42 ± 6.87	76.25 ± 13.56	64.45 ± 9.58	0.318
IL-8	186.23 ± 19.68	235.56 ± 31.56	206.72 ± 24.56	0.411
IL-12	134.15 ± 12.14	186.84 ± 24.51	164.56 ± 19.45	0.180
GM-CSF	135.12 ± 11.58	169.56 ± 20.45	132.45 ± 12.48	0.188
TGF-*β*1	817.24 ± 38.42	728.63 ± 56.25	795.42 ± 41.56	0.383
TNF-*α*	2.87 ± 0.17^b^	9.54 ± 2.14^a^	3.57 ± 1.06^b^	0.005
IL-10	0.38 ± 0.08^b^	0.75 ± 0.10^a^	0.51 ± 0.10^a, b^	0.034
IFN-*γ*	0.10 ± 0.02	0.19 ± 0.09	0.15 ± 0.09	0.697

Values are the mean ± SEM, *n* = 8 per treatment group. ^a-c^Mean values sharing different superscripts within a row differ (*p* < 0.05).

**Table 5:** Serum concentrations of D-lactate and diamine oxidase in piglets.

Item	Basal diet	Basal diet + diquat	EUF diet + diquat	*p* value
Day 14				
D-lactate (mmol/L)	0.74 ± 0.03^b^	0.98 ± 0.08^a^	0.82 ± 0.04^a, b^	0.016
Diamine oxidase, mg/ml	41.16 ± 3.36^b^	54.21 ± 3.67^a^	45.87 ± 2.14^a, b^	0.024
Day 21				
D-lactate, (mmol/L)	0.25 ± 0.02	0.31 ± 0.04	0.29 ± 0.05	0.546
Diamine oxidase, mg/ml	36.15 ± 1.15	39.36 ± 3.10	37.56 ± 2.16	0.615

Values are the mean ± SEM, *n* = 8 per treatment group. ^a-b^Mean values sharing different superscripts within a row differ (*p* < 0.05).

**Table 6:** Jejunal and ileal morphology in piglets.

Item	Basal diet	Basal diet + diquat	EUF diet + diquat	*p* value
Day 14				
Jejunal villous height (*μ*m)	297.52 ± 7.21^a^	226.53 ± 21.08^b^	306.21 ± 27.35^a^	0.026
Jejunal crypt depth (*μ*m)	93.23 ± 7.14	129.25 ± 18.35	101.25 ± 16.24	0.217
Jejunal villous height: crypt depth	3.19 ± 0.23^a^	1.75 ± 0.31^b^	3.02 ± 0.17^a^	<0.001
Ileal villous height (*μ*m)	240.32 ± 10.56^a^	164.38 ± 27.32^b^	237.32 ± 15.25^a^	0.016
Ileal crypt depth (*μ*m)	69.15 ± 3.25^b^	86.15 ± 5.43^a^	72.43 ± 9.15^a, b^	0.043
Ileal villous height: crypt depth	3.48 ± 0.17^a^	1.91 ± 0.38^b^	3.28 ± 0.28^a^	0.002
Day 21				
Jejunal villous height (*μ*m)	246.32 ± 12.23	216.32 ± 32.12	242.43 ± 15.35	0.578
Jejunal crypt depth (*μ*m)	114.26 ± 9.47	144.39 ± 21.26	113.21 ± 12.74	0.284
Jejunal villous height: crypt depth	2.16 ± 0.24	1.50 ± 0.41	2.14 ± 0.27	0.265
Ileal villous height (*μ*m)	189.32 ± 12.23	154.16 ± 26.36	179.94 ± 13.45	0.084
Ileal crypt depth (*μ*m)	102.13 ± 5.13	125.34 ± 19.44	101.64 ± 11.21	0.372
Ileal villous height: crypt depth	1.85 ± 0.31	1.23 ± 0.31	1.77 ± 0.16	0.232

Values are the mean ± SEM, *n* = 8 per treatment group. ^a-b^Mean values sharing different superscripts within a row differ (*p* < 0.05).

**Table 7:** Histopathological grading of the jejunum, ileum, and colon in piglets.

Item	Basal diet	Basal diet + diquat	EUF diet + diquat	*p* value
Day 14				
Jejunum	2.25 ± 0.31^c^	12.13 ± 0.44^a^	6.13 ± 0.44^b^	<0.001
Ileum	3.25 ± 0.45^c^	9.13 ± 0.55^a^	5.00 ± 0.46^b^	<0.001
Anterior colon	4.00 ± 0.38^c^	10.50 ± 0.76^a^	6.50 ± 0.57^b^	<0.001
Posterior colon	4.38 ± 0.63^c^	11.00 ± 0.65^a^	7.75 ± 0.75^b^	<0.001
Day 21				
Jejunum	3.00 ± 0.38	4.50 ± 0.46	3.25 ± 0.49	0.059
Ileum	3.75 ± 0.49	3.88 ± 0.52	3.75 ± 0.49	0.980
Anterior colon	4.50 ± 0.57	4.75 ± 0.53	4.13 ± 0.64	0.748
Posterior colon	4.25 ± 0.65	5.38 ± 0.71	5.00 ± 0.73	0.519

Values are the mean ± SEM, *n* = 8 per treatment group. ^a-c^Mean values sharing different superscripts within a row differ (*p* < 0.05).

## Abbreviations

CAT: Catalase
DAO: Diamine oxidase
EUF: *Eucommia ulmoides* flavones
GM-CSF: Granulocyte macrophage colony stimulating factor
GSH-Px: Glutathione peroxidase
H_2_O_2_: Hydrogen peroxide
IFN-*γ*: Interferon-gamma
IL: Interleukin
MDA: Malondialdehyde
MPO: Myeloperoxidase
NF-*κ*B: Nuclear factor kappa-beta
NO: Nitric oxide
RNS: Reactive nitrogen species
ROS: Reactive oxygen species
SOD: Superoxide dismutase
T-AOC: Total antioxidant capacity
TGF-*β*1: Transforming growth factor-beta 1
TNF-*α*: Tumor necrosis factor.
